# Gene Therapy with Helper-Dependent Adenoviral Vectors: Current Advances and Future Perspectives

**DOI:** 10.3390/v2091886

**Published:** 2010-09-03

**Authors:** Francesco Vetrini, Philip Ng

**Affiliations:** Department of Molecular and Human Genetics, Baylor College of Medicine, Houston, TX, USA; E-Mail: vetrini@bcm.edu

**Keywords:** Helper Dependent Adenovirus, liver transduction, lung transduction, innate and adaptive immune response

## Abstract

Recombinant Adenoviral vectors represent one of the best gene transfer platforms due to their ability to efficiently transduce a wide range of quiescent and proliferating cell types from various tissues and species. The activation of an adaptive immune response against the transduced cells is one of the major drawbacks of first generation Adenovirus vectors and has been overcome by the latest generation of recombinant Adenovirus, the Helper-Dependent Adenoviral (HDAd) vectors. HDAds have innovative features including the complete absence of viral coding sequences and the ability to mediate high level transgene expression with negligible chronic toxicity. This review summarizes the many aspects of HDAd biology and structure with a major focus on *in vivo gene* therapy application and with an emphasis on the unsolved issues that these vectors still presents toward clinical application.

## 1. Introduction

Adenovirus (Ad)-derived gene therapy vectors have been the focus of considerable interest for their potential application as a delivery vehicle for human gene therapy. Even though these vectors were initially conceived for the treatment of genetic disorders, so far the majority of their clinical application has been for cancer treatment. Some of the most important aspects of Ad-derived vectors that have gained the attention of the gene therapy community include: 1) the ability to infect with high efficiency a variety of both quiescent and proliferating cell types, 2) Ad vectors can be easily grown to very high titer, allowing the experimenter to transduce a large number of cells and/or tissue target of large animals, and 3) the absence of vector genome integration thereby reduces the likelihood for germ-line transmission and insertional mutagenesis, which represents an important safety feature [1] for human clinical applications. First generation adenoviral (FGAd) vectors are rendered replication-deficient by the deletion of the viral early region (E1). However, leaky expression of viral genes from the vector backbone is still present, leading to loss of transgene expression due to an adaptive cellular immune response against the transduced cells and chronic toxicity. Further deletion in the vector backbone (E2, E4) still allows for the low level expression of viral genes and does not preclude an immune reaction towards transduced tissues [2,3]. In contrast, helper-dependent adenoviral (HDAd) vectors are devoid of all viral coding sequences, and have shown tremendous potential for the treatment of genetic disease, allowing for persistent transgene expression for years in the apparent absence of any type of adaptive immune response and chronic toxicity [4]. HDAds can mediate high efficiency transduction, do not integrate in the host genome, and have a large cloning capacity of up to 37 kb, which allows for the delivery of multiple trangenes or entire genomic loci, or large *cis*-acting elements to enhance or regulate tissue-specific transgene expression (for example, inclusion of the large control elements of the human cytokeratin 18 gene for specific expression in the epithelium [5] or inclusion of the human apolipoprotein E Hepatic Control Region for enhanced liver specific expression [6]). This review will focus on the general features of the HDAd vectors and the most recent advance in the clinical application of these vectors.

## 2. Adenoviruses

**Figure 1.**
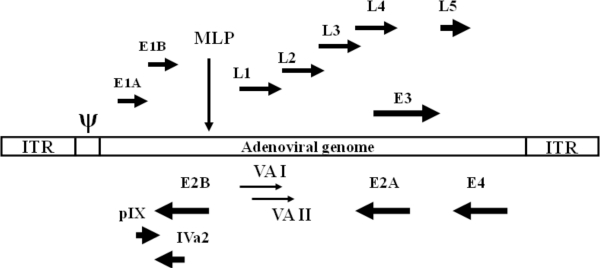
Transcription map of human adenovirus serotype 5. The 100 map unit (∼36 kb) genome is divided into four early region transcription units, E1–E4, and five families of late mRNA, L1–L5, which are alternative splice products of a common late transcript expressed from the major late promoter (MLP) located at 16 map units. Four smaller transcripts, pIX, IVa, and VA RNA’s I and II, are also produced. The 103 bp inverted terminal repeats (ITRs) are located at the termini of the genome and are involved in viral DNA replication, and the packaging signal (ψ) located from nucleotides 190 to 380 at the left end is involved in packaging of the genome into virion capsids. Other late transcripts include the RNA polymerase III.

Adenoviruses (Ad) are non-enveloped double stranded DNA viruses of 60–110 nm in diameter. During natural infection, the main target is epithelial cells of the respiratory and gastrointestinal tract. Ad can cause relatively mild, self-limiting diseases of the upper respiratory tract, gastroenteritis, or conjunctivitis in some cases but most infections are asymptomatic in immunocompetent individuals [7–11]. Importantly, adenoviruses have not been associated with any neoplastic disease in humans [7]. Amongst the ∼50 serotypes of human Ad, the most extensively characterized are serotypes 2 (Ad2) and 5 (Ad5) belonging to subgroup C. The 36 kb genome of Ad 2 and Ad5 is flanked by *cis*-acting inverted terminal repeats (ITRs), which are required for viral DNA replication in *cis*. A *cis*-acting packaging signal (Ψ), required for the encapsidation of the Ad genome, is located near the left ITR (relative to the conventional map of Ad) (Figure 1). The Ad genome comprises two set of genes (Figure 1): the early region genes, E1A, E1B, E2, E3, and E4, are transcribed before DNA replication and the late region genes, L1 to L5, are transcribed and expressed at high levels after the initiation of DNA replication. The E1A transcription unit encodes two major E1A proteins that are involved in transcriptional regulation of the virus and stimulation of the host cell to enter an S phase-like state. The two major E1B proteins are involved in stimulation of viral mRNA transport, blocking E1A-induced apoptosis and blocking host mRNA transport. The E2 region can be divided into two sub-regions: E2a encodes the DNA-binding protein and E2b encodes the viral polymerase and terminal protein precursor. The E3 region encodes at least seven proteins, most of which have immunomodulatory functions and are specifically involved in host immune evasion. The E4 region encodes at least six proteins involved in DNA replication, enhancement of the late gene expression, and decrease host protein synthesis. Alternative splicing of a single transcript, referred to as late region genes, gives rise to all the mRNA encoding virion structural proteins. The expression of late region genes is regulated by a common major late promoter (MLP).

Infection of the host cell is a two stage process involving an initial binding of the Ad fiber protein with CAR (coxsackie-adenovirus receptors) on the cell surface [9–12]. Then a secondary interaction occurs between the arginine-glycine-aspartic acid (RGD) motif present on the virion penton base and α_v_β_3_ and α_v_β_5_ integrins on the host cells, which in turn initiates receptor-mediated endocytosis via clathrin-coated pits [12–15].The efficiency of infection, which is dependent on Ad binding and entry, is directly related to the level of primary and secondary receptors on the cell surface [12,14]. After Ad internalization, the virion escapes from the early endosome into the cytosol prior to lysosome formation [16,17]. During the translocation along the microtubule network toward the nucleus, the virion is disassembled and the DNA is released into the nucleus [18]. Once in the nucleus, viral DNA replication (beginning at 6–8 h postinfection) and assembly of progeny virion occur. The entire life cycle takes about 24–36 h and generates about 10^4^ virions per infected cell.

## 3. Early-generation Adenoviral vectors

The basic notion that permitted the construction of first generation of Ad vectors (FGAd) is based on the fact that up to 10% of Ad viral DNA molecules become circularized following infection of mammalian cells [19]. Therefore, the entire Ad genome can be manipulated as an infectious bacterial plasmid by using standard molecular biology techniques. Indeed, the Ad genomic plasmid could be easily and stably propagated in *E. coli* and was capable of generating infectious viruses following transfection into permissive mammalian cells. The earliest version of FGAd vectors were prepared by substituting E1 region of Ad5 with a transgene. This type of vector can deliver transgenes up to 5 kb and cannot replicate in transduced cells, because they lack the E1 region which controls the transactivation of genes involved in viral replication. An E1 deficient (ΔE1) vector must be propagated in a permissive cell line, engineered to provide the E1 functions in *trans.* While the deletion of E1 in FGAd results in a replication-defective vector, nonetheless E2, E3, and E4 promoters are still active and result in viral low level DNA replication and expression of viral genes, especially at high doses of infection [20,21]. This leaky expression of viral proteins precludes long-term transgene expression *in vivo* due to the strong cytotoxic immune reaction mounted against the transduced cells, resulting in the extinction of transgene expression within few weeks [22–24]. Therefore, in order to minimize the immune reaction toward the viral antigens, Ad vectors with further deletion of essential viral genes have been generated. Second generation or multiply-deleted Ad vectors carrying mutation or alteration in E2 or E4 regions in addition to E1 were constructed in parallel with the development of more complex producer cell lines capable of supporting their replication by *trans*-complementing the additional deletions [20]. However, viral coding sequences still remain and therefore so do the potential for their expression and related toxicity. The advantages of multiply-deleted Ad vectors over FGAd remain controversial as some studies show them to be superior in terms of toxicity and duration of transgene expression [25,26] while some others do not [22,27–29].

## 4. HDAd

**Figure 2.**
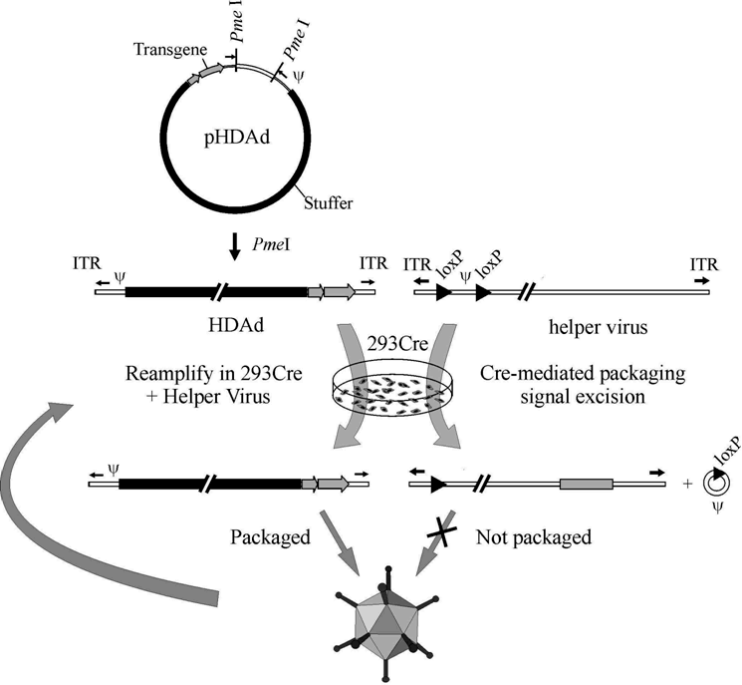
The Cre/loxP system for generating HDAds. The HDAd contains only ∼500 bp of *cis*-acting Ad sequences required for DNA replication (ITRs) and packaging (ψ); the remainder of the genome consists of the desired transgene and non-Ad *stuffer* sequences. The HDAd genome is constructed as a bacterial plasmid (pHDAd) and is liberated by restriction enzyme digestion (e.g., *Pme*I). To rescue the HDAd, the liberated genome is transfected into 293 cells expressing Cre and infected with a helper virus bearing a packaging signal (ψ) flanked by loxP sites. Cre-mediated excision of ψ renders the helper virus genome unpackageable, but still able to provide all of the necessary *trans*-acting factors for propagation of the HDAd. The titer of the HDAd is increased by serial coinfections of 293Cre cells with the HDAd and the helper virus.

The liver toxicity and the cellular immune response triggered by early generation Ad vectors constituted a great incentive for the construction of a new type of vector completely devoid of all adenoviral genes, referred to as Gutless Adenovirus or Helper-dependent Adenoviral (HDAd) vector. HDAds are constructed by removing all viral sequences from the vector genome except the packaging sequence and inverted terminal repeats, thereby eliminating the issue of residual viral gene expression associated with early generation Ad vectors. Currently, the most efficient method for generating HDAd is the Cre/loxP system; this method was the result of pioneering work by Frank Graham and co-workers in 1996 [30]. In this system, the HDAd genome is constructed in a bacterial plasmid and contains the expression cassette of interest and ∼500 bp of *cis*-acting Ad sequences necessary for vector DNA packaging (Ψ) and replication (ITRs). In order to obtain efficient packaging, the inclusion of stuffer DNA is required to maintain the size of the vector within appropriate limits. The size of HDAd vectors below ∼27 kb undergo DNA rearrangement to increase the size of the genome to 27 to 38 kb [31,32]. To convert or rescue the “plasmid form” of HDAd to the “viral form”, the plasmid is first digested with the appropriate restriction enzyme to liberate the HDAd genome from the bacterial plasmid sequences. Then, 293 cells expressing Cre are transfected with the linearized HDAd genome and subsequently infected with a helper virus. The helper virus is a FGAd bearing a packaging signal flanked by loxP sites in its genome and trans-complement the replication and encapsidation of the HDAd genome. Following the infection of 293 Cre cells, the packaging signal is excised from the helper virus genome by Cre-mediated site-specific recombination between the two loxP sites, and this precludes the packaging of the helper virus (Figure 2). Further improvements to this system have been made, which has permitted large scale manufacture of high quality HDAd with extremely low levels of helper virus contamination [33] for large animal preclinical studies [34–39]. Indeed, using this improved system [33], cGMP grade HDAd has been manufactured to treat patients with anemia with very encouraging results [159]. In addition to the Cre/loxP system, the analogous FLP/frt systems have also been developed for the production of HDAds [40,41] although refinement of these systems have not progress as far.

One of the most attractive features of HDAd vectors is the long term expression of the transgene, which will be discussed in detail below. In this regard, Jager *et al*. have recently shown that compared to plasmid non viral DNA, the HDAd genome, which exists predominantly as linear monomers in the nucleus of the cell, seems to be more stable [42,43]. Several potential mechanism that could explain the persistence of HDAd genome have been proposed by the authors: HDAd genomes 1) replicate episomally, 2) possess a nuclear retention signal, 3) circularize, 4) integrate or 6) associate with histones [43]. Further investigations are needed to fully elucidate this phenomenon to permit the manipulation of HDAd genome in order to render it even more stable and safe. Some other important considerations concerning HDAd construction and production are the vector genome size for encapsidation and the composition of the stuffer DNA. Vector genome size below 27Kb were inefficiently packaged and undergo DNA rearrangement to produce larger genome closer to the wild type Ad5 genome size (approximately 30Kb) [31]. Vector genome sizes above the maximum packaging capacity were not efficiently packaged [31]. Packaging of viral DNA that is too small results in destabilized virions, which would result in a growth disadvantage [44]. Since the minimal Ad *cis*-acting sequences and the transgene are usually below the minimal size required for efficient packaging, the inclusion of a DNA stuffer is needed. The choice of DNA stuffer influences the vector stability, replication efficiency and *in vivo* performance [32,45]. A recent report by Smith *et al*. showed that HDAd virion with a genome size of ∼30 kb were 100-fold more sensitive to heat inactivation than viruses with larger genome sizes (>36 kb) and that increasing the genome size significantly improved heat stability, but these smaller genome vectors were equally stable at physiologic temperatures [46]. It has been shown HDAd with bacteriophage lambda DNA as stuffer resulted in a decrease in the level and duration of transgene expression secondary to a cytotoxic T lymphocyte (CTL) immune response directed against the peptides derived form the viral backbone [32]. Interestingly, substituting the lambda stuffer with eukaryotic DNA sequences such as human hypoxanthine-guanine phosphoribosyltransferase gene, resulted in significantly higher transgene expression in the absence of CTL immune response, demonstrating the influence of the stuffer composition on the immunological properties of HDAd [32].

## 5. HDAd *in vivo* studies

### 5.1. Liver directed gene therapy

The liver is the key organ in many metabolic processes and is the affected organ in numerous inherited metabolic disorders. Gene therapy strategies aimed at targeting the liver offer several advantages: the fenestrated structure of its endothelium permits exposure of the parenchymal cells to systemically delivered vector, transduced hepatocytes permit the secretion of the vector encoded transgene product, making the liver a factory for production and secretion of therapeutic proteins. To date, numerous examples of *in vivo* liver-directed gene therapy for disease models using HDAd have been reported. In general, all of these studies have demonstrated the tremendous therapeutic potential of HDAd vectors.

Atherosclerosis is a common and complex pathological process characterized by intimal foam cell accumulation and extracellular matrix deposition in medium and large-sized arteries. A practical model for this disease is the apolipoprotein E knockout (apoE-/-) mouse [47,48]. To evaluate the efficacy of HDAd *versus* FGAd, hypercholesterolemic apoE-/- mice were injected with HDAd or FGAd encoding the mouse apoE cDNA (HD-Ad5-cE or FG-Ad5-cE) or a HDAd bearing the mouse genomic apoE locus (HD-Ad5-gE) [47]. Intravenous injection of apoE deficient mice with FG-Ad-cE or HD-Ad-cE resulted in a rapid increase of ApoE protein in the serum with a concomitant fall in plasma cholesterol levels to within normal range. However, in sharp contrast to FG-Ad-cE, in which the cholesterol level returned to the pathological levels within about 14 weeks, normalization of cholesterolemia by HD-Ad-cE lasted about a year before gradually increasing. Intravenous injection of HD-Ad5-gE resulted in complete and immediate drop of plasma cholesterol to normal levels, staying within the normal range for the rest of the natural lifespan of the animal (about 2.5 years). Further analysis by quantitative morphometry of the aorta showed the absence of atherosclerotic plaques at two years after the HDAd injection. These results demonstrated the superiority of HDAd over FGAd and also that genomic based transgenes may be more effective than cDNA based transgenes.

Simple re-administration of the vector when the transgene expression fades is precluded by neutralizing anti-Ad antibodies generated following the first administration. Indeed, mice previously injected with HD-Ad5-cE could not be successfully re-injected again. One potential solution is to use a different HDAd serotype for re-injection. In order to evaluate this strategy, Kim *et al*. generated a serotype 2 version of HD-Ad5-gE (HD-Ad2-gE) [47] and showed that it could be successfully administered to mice previously treated with the serotype 5 (HD-Ad5-gE) to re-gain the expression of ApoE protein and lower plasma cholesterol levels. In this study, the authors also assessed the toxicity associated with the vectors and found that the FG-Ad5-cE vector resulted in significant hepatotoxicity as indicated by significant elevation of AST/ALT (>10- to 20-fold), whereas no such evidence of toxicity was associated with any of the HDAd vectors, even after a second administration with the serotype 2 HDAd [47]. In conclusion, this study demonstrated that: 1) a single intravenous injection of HDAd results in life-long expression of the therapeutic transgene and permanent phenotypic correction of a genetic disease, 2) the large cloning capacity of the HDAd allows for the delivery of the transgenes in their native genomic context, which resulted in superior kinetics and duration of the expression, 3) negligible toxicity was associated with HDAd administration, 4) administration of an alternative serotype HDAd is effective at circumventing the humoral immune response generated by the initial treatment.

Crigler-Najjar syndrome type I is a severe inborn error of bilirubin metabolism due to mutations in the uridinediphosphoglucuronate glucuronosyltransferase (UGT1A1) gene. Affected patients have increased serum bilirubin levels which may be life-threatening. Because of the risk of brain damage, patients are often treated with liver transplantation [49]. Toietta *et al*. [50] showed that a single systemic injection of HDAd expressing UGT1A1 in Crigler-Najjar rats resulted in life-long expression of UGT1A1 and permanent phenotypic correction of the hyperbilirubinemia.

HDAd liver-mediated gene transfer can be considered an important tool for numerous diseases beyond monogenic disorders. An interesting application of HDAd for the treatment of diabetes mellitus has also been reported. In this study, two HDAds, one expressing *Neurod1* (a transcription factor expressed in developing and adult β-cells of the pancreas) and another expressing *betacellulin* (a growth factor specific for β-cells), were co-injected into diabetic mice [51]. The results showed a normalization of glucose levels for the duration of the experiment (at least 120 days). This study showed that the manipulation of pancreatic precursor present in the liver, through the expression of critical factors for their development/differentiation by HDAd-liver directed gene therapy, can efficiently revert the diabetic phenotype in an animal model.

An application of HDAd liver-directed gene therapy aimed at silencing a target gene by using short hairpin RNA (shRNA) has been recently reported. In this study, HDAd expressing shRNA directed against a transcription factor (SREBP1), which is upregulated in obese mice, showed a reduction in body weight [52]. In another application, the authors demonstrated the ability of HDAd-shRNA to silence the expression of specific mouse genes by approximately 75–90% [53]. It is remarkable that in contrast with previous studies showing severe toxicity and lethality following administration of AAV encoding shRNA, the HDAd expressing shRNA was well tolerated and showed only mild or low hepatotoxicity [53]. One of the major issues encountered with AAV shRNA vectors is the saturation of the exportin-5 pathway, which shuttles cellular micro-RNA (mi-RNA) from the nucleus to the cytoplasm, and is thought to be involved in the observed toxicity with AAV [54]. In contrast, a similar mechanism was not seen with HDAd expressing shRNA [52]. These encouraging results with HDAd-shRNA may pave the way to a variety of applications involving the silencing of dominant mutations causing genetic and acquired diseases.

The impressive duration of transgene expression observed in rodents has been recapitulated in large animal models. In one study, two hemophilia B dogs injected 3 × 10^12^ vp/Kg of HDAd expressing the canine factor IX resulted in a sustained phenotypic improvement of the bleeding diathesis for the duration of the experiment of at least 604 and 446 days [35]. Another group reported the correction of hemophilia A in dogs for several months with a minimal observed liver toxicity [55]. Studies conducted in nonhuman primates convincingly demonstrated that HDAd was superior to FGAd with respect to duration of transgene expression and liver toxicity. In one of these studies, three baboons were intravenously injected with HDAd expressing hAAT (human α-1 anti-tripsin) [56]. hAAT expression persisted for more than one year in two of the three animals. It was significant that no abnormalities in blood cell counts and liver enzymes were observed in these three baboons. In contrast, FGAd expressing hAAT generated a cellular immune response directed against the transduced cells causing loss of transgene expression. It was demonstrated that viral protein expressed from the FGAd viral backbone contributed to this loss [56]. These and other studies provide compelling evidence for using HDAd to treat genetic disorders.

#### 5.1.1. Nonlinear dose-response to hepatocyte transduction

In the aforementioned studies, relatively high vector doses were used to achieve efficient hepatic transduction. Indeed, there is a non linear dose-response to the vector with high doses required for efficient hepatocyte transduction. This results in widespread vector dissemination as well as dose dependent activation of the innate immune response, resulting in acute toxicity with potentially severe and lethal consequences. This acute activation of the innate immune response is characterized by high levels of serum inflammatory cytokines and chemokines within a few hours post-injection. Kupffer cells (KC) in the liver play a major role in this non-linear dose-response by taking up the majority of the viral particles that reach the liver, precluding hepatocyte transduction. Furthermore, systemic administration of Ad vectors likely results in widespread transduction of a large number of extrahepatic tissues, which also contribute to inefficient hepatocytes transduction [57,58].

Hepatocyte transduction *in vivo* doses not seem to be mediated by CAR receptor-and/or integrin but involve the interaction of Ad vector with bloodborne components such as coagulation factors and complement factors. Recent studies showed that pretreatment of mice with warfarin before Ad5 vector injection, abrogates liver transduction. Warfarin, which prevents the maturation and secretion of functional vitamin K–dependent coagulation factors, inactivates several proteins belonging to the coagulation cascade pathway (factors II, VII, IX and X, anticoagulant protein C) and presumably inhibits the interaction between these factors and Ad5 vectors. It has been proposed that coagulation proteins act as a bridge between hepatocytes and Ad5 vector [59–63]. Recently, the mechanism by which factor X interact with Ad5 and promote the transduction of hepatocytes has been unraveled [64]. Electron cryomicroscopy studies determined that factor X binds within cavities formed by trimeric hexon proteins and involves interaction with the Ad5 hexon hypervariable regions [64]. This interaction promotes the binding of the Ad5–factor X complex to cellular heparan sulfate proteoglycans (HSPGs) and consequently, utilization of HSPGs as receptors by the Ad5–factor X complex, and not CAR. This seems to be important for liver transduction by Ad5 after systemic administration *in vivo.* Notably, in contrast with the species C serotypes Ad5 and Ad2, which have been shown to transduce hepatocyte after systemic injection, species B Ad35 and species D Ad26 have a weak if not absent binding to factor X and do not transduce the liver [65]. However, it is now clear that despite the fact that factor X facilitates Ad5 entry into hepatocytes, it is not required for trapping of vectors in the liver. Several other mechanisms contribute to the adenovirus sequestration by the liver [57], including: a) trapping of the virus by liver Kupffer cells and sinusoidal cells [66–68], b) Ad5 penton RGD motif-mediated interactions with liver endothelial cells and hepatocytes with consequent retention of the viral particle in the space of Disse [57], and platelets in blood may contribute significantly to sequestration in the liver reticulo-endothelial system [69]. Di Paolo *et al*. [57] showed that simultaneous treatment of mice with clodronate liposomes, which deplete Kupffer cells and warfarin results in only a minimal reduction in sequestration in the liver. In addition, antibodies both specific and non specific for Ad, may also play a significant role in the non linear dose-response. Studies have shown that the threshold effect to hepatic transduction by Ad is reduced in antibody deficient Rag-1 and μMT mice [66,70]. One possible interpretation is that opsonization of the virion by antibodies may enhance the efficiency of Fc-receptor mediated uptake by Kupffer cells.

#### 5.1.2. Immunobiology of HDAd and pathogenesis of the acute toxicity

Adenoviruses, along with other microorganisms, following infection, are subject to the host immune response, which has evolved and adapted as a defense to fight off the pathogen invasion. Although early generation of Ad vectors are unable to replicate their genome, they still share several features with the wt Ad counterpart. Therefore, systemic delivery of these vectors in animals or humans triggers an immune reaction, with potentially severe consequences for the host. Systemic delivery of FGAd vectors is known to induce a potent cellular adaptive immune response against the viral components and the transgene, and a non-specific but acute innate immune response. The immune reaction following FGAd administration in rodents or non human primates has been well characterized and comprises two phases. The first phase is mediated by the capsid and the second by expression of viral genes. After systemic injection of FGAd, the earliest phase occurs within minutes, is characterized by acute production of pro-inflammatory cytokines and chemokines such as Interleukin-6/12, RANTES (regulated upon activation, normal T cell expressed and secreted), macrophages chemoattractant protein-1 (MCP-1), interferons and others, and its severity is dose-dependent [34,71] .The high level of serum inflammatory cytokines and chemokines results in systemic inflammatory response syndrome and multiple organ disfunction syndrome [4]. The second (e.g. chronic) phase lasts for days and occurs only with FGAd, and is associated with CTL mediated clearance of vector-transduced cells. It has been demonstrated that the first phase does not require transcription of viral genes but is initiated by the interaction of the viral capsid with the cells of the immune system [72]. Indeed, this acute toxicity is lethal in non human primates with both FGAd and HDAd at relatively high doses [34,71]. The innate immune response following intravascular administration of FGAd and HDAd vectors appears identical, and is complex and multifactorial.

The important role of pattern recognition receptor (PRR), such as Toll-like receptors (TLR)-9 and TLR-2 in triggering the innate immune response has recently emerged [73]. TLR-2 is associated with the cellular membrane and is probably involved in the recognition of the capsid proteins [74,75]. However, the adenoviral ligand to TLR-2 has yet to be identified. TLR-9 is an endosomal receptor and recognizes the DNA component of the Ad vectors [76–78]. Ad vector interaction with these two sensor-receptors engages a complex intracellular pathways through the activation of myeloid differentiation primary response gene 88 (MyD88) that culminate in the massive production of cytokine and chemokines, interferon (IF)-α and β and triggers dendritic cells (DCs) maturation and development of T-cell and B-cell responses against the Ad vector components [79]. The type I-IFs (α and β) activate natural killer (NK) cells and have a predominant role in the subsequent regulation of the innate immune response machinery against the vector [80]. Subsequent activation of chemoattractant protein (MIP-2), Interleukin-1 and tumor necrosis factor contribute to leukocytes infiltration in the target tissue. NK cells activation releases several cytokines and promotes an adaptive immune response to the vector [81].

Importantly, Ad vectors elicit the innate immune response either through MyD88/TLR or in an independent pathway depending on the cell type [73,82]. For example, DCs uses both MyD88 and TLR-9 for cytokine production, whereas activation of peritoneal macrophages and subsequent release of cytokines is independent of MyD88/TLRs system [73]. In the MyD88/TLR independent activation of innate immune response, double stranded viral DNA is recognized by a cytosolic molecular complex known as the inflammasome. The inflammasome consists of NALP3 and ASC adaptor protein complex, which induces maturation of pro-IL-1β in macrophages after the interaction with the viral DNA. This mechanism seems to be a key event in the innate immune response to Ad vectors and other DNA viruses. Another recent study by Di Paolo *et al*. identifies IL-1α-IL-1receptor-I (IL-1R-1) as a key pathway allowing for the activation of pro-inflammatory responses to the virus in macrophages, independently of recognition of the virus-associated nucleic acid by intracellular PRR [83]. The authors showed that the IL-1α-mediated response requires a selective interaction of virus RGD motifs with macrophage β3 integrins in response to Ad vector, leading to production of inflammatory cytokines and chemokines [83].

As already mentioned in the previous section, Ad vectors can also interact with various bloodborne components, which affects the efficiency of liver transduction and the tissue biodistribution. Moreover, blood factors including complement protein, coagulation system and both neutralizing and non-neutralizing antibodies may contribute to the acute toxicity. Ad has been shown to bind and activate the complement components including C3 and C4BP in the classical and alternative complement pathways [84,85]. Complement and antibody interactions with Ad vectors result in an acute response with secretion of cytokines and chemokines [84]. These latter interactions promote the adhesion and migration of infiltrating leukocytes and platelet aggregation. Thrombocytopenia is caused by interaction between adenoviral particles and the coagulation system, resulting in formation of platelet-leukocyte aggregates [81,86].

It is worth mentioning that recombinant AAV (Adeno associated virus) vectors, like HDAds, are devoid of all viral gene sequences. In preclinical studies for liver-directed gene therapy, AAV vectors, similarly to HDAd, have shown to provide long-term transgene expression in mouse, dog and non human primates [87,87–91]. Despite these encouraging results, a human clinical trial for hemophilia B with AAV2 showed an unexpected outcome: subjects of the study developed transient elevation of liver enzymes and loss of FIX transgene expression after a few weeks secondary to a CTL immune response mediated by the re-activation of pre-existing AAV capsid-specific CD8+T memory cells [92]. This CTL response occurred in the absence of viral gene transcription and is due to a cross-presentation of the AAV capsid input proteins through the major histocompatibility complex (MHC)-I [93]. Based on this experience with AAV, it will be important to determine whether a similar immune reaction will occur in humans following transduction by HDAd. Previous *in vitro* studies suggested that the induction of anti-Ad CTL is similar with either FGAd and HDAd [94] and that *de novo* expression of viral genes from Ad backbone is not a pre-requisite for elicitation of CTL immune response [95]. In addition, studies performed in naïve mice indicated that the key difference between FGAd and HDAd is that both initiate anti-Ad CTL against tranduced hepatocytes, but only FGAd-transduced cells are cleared, probably due to the leaky expression of viral genes [3]. Finally, a recent study showed that pulmonary delivery of HDAd in mice results in eliciting CD8+ T cells that are able to trigger an adaptive immune response against the transduced cells even at low dose of vector [96,97]. However, comparative analysis between FGAd and HDAd are needed to address the role of the dose, species, and pre-immunization status of the host. Since the majority of the human have pre-existing immunity to Ad5, it may be necessary to develop strategies to prevent adaptive immune response against HDAd transduced cells before clinical translation. .

#### 5.1.3. Overcoming the threshold effect and the acute toxicity

**Figure 3.**
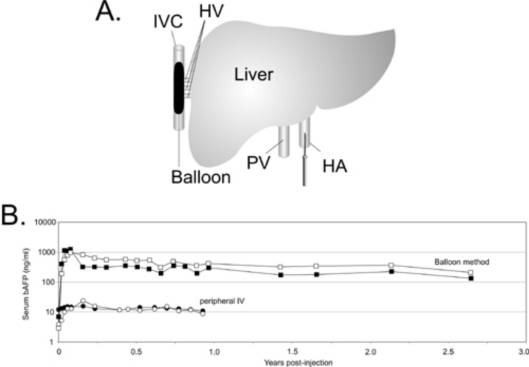
**(A)** A sausage-shaped balloon catheter is positioned in the inferior vena cava (IVC) under fluoroscopic guidance. Inflation of the balloon results in hepatic venous outflow occlusion from the hepatic veins (HV). The HDAd is administered by injection through a percutaneously positioned hepatic artery (HA) catheter. **(B)**. Serum levels of the reporter baboon α-fetoprotein (bAFP) following administration of 3x10e10 vp/kg of a HDAd expressing bAFP into baboons using the balloon method described above (squares) or by simple peripheral intravenous injection (circles). The balloon method of vector delivery yielded up to 80-fold higher level of transgene expression compared to peripheral intravenous injection of vector, and transgene expression persisted at high levels for at least 2.5 years.

Strategies to overcome the hepatic threshold effects of transduction while reducing the systemic vector dissemination are highly desirable to improve the therapeutic index of HDAd for liver-directed gene therapy. Several approaches have been investigated and some are currently under investigation. Because the severity of the acute response is dose-dependent, some of these approaches are aimed at preferential targeting of the vector to hepatocyte thereby allowing for the use of lower vector doses. For example, injection of HDAd directly in the surgically isolated liver of nonhuman primates was shown to achieve higher efficiency hepatic transduction with reduced systemic vector dissemination, and stable, multi-year transgene expression without chronic toxicity [11]. However, the invasiveness of this method makes it clinically unattractive. Several studies suggested that the liver microarchitecture plays a prominent role in the liver transduction efficiency by Ad vectors. The size of the sinusoidal endothelial fenestrae (SEF) of the liver, which is approximately 100 nm, plays a critical role in hepatocyte transduction by Ad since the Ad virion size is ≥100 nm. It has been shown that there is a direct correlation between the size of the SEF and the efficiency of hepatocyte transduction following systemic administration of Ad [98,99]. Therefore, physical or chemical methods to enlarge SEF diameter could have a positive impact on the therapeutic index of Ad vectors, by increasing hepatocyte transduction with lower vector doses thereby reducing the acute toxicity. The size of the SEF can be enlarged by means of drugs such as Na-decanoate or N-acetylcysteine [98], combined with transient liver ischemia or pretreatment with the neuropeptide vasoactive intestinal peptide (VIP) [100] among others. Another interesting approach to enlarge the SEF in rodents is increasing the intrahepatic pressure by hydrodynamic injection (h.i.), a technique which involves the rapid tail vein injection of large volumes [101–103]. In mice, h.i. of HDAd results in improved hepatocyte transduction with concomitant reduction of the systemic dissemination of the vector [102]. However, further studies are necessary to determine the real clinical potential of these SEF enlarging drugs, and h.i. as performed in rodents is not suitable for human application due to the requirement of rapid, large volume injection. Interestingly, h.i. studies suggested that high efficiency of hepatic transduction does not, at least alone, necessarily provoke a potent inflammatory response and that systemic dissemination of the vector may play a major role in the severity of the inflammatory response [102]. A clinically attractive method of delivering HDAd which mimics h.i. has been developed for large animals by using balloon occlusion catheters [37]. In this approach, referred as pseudo-hydrodynamic injection, hepatic venous outflow is occluded using two balloon occlusion catheters percutaneously placed in the inferior vena cava (IVC), above and below the hepatic veins (HV). An increase in intrahepatic pressure with this method is achieved because blood that enters the liver from the hepatic artery (HA) and portal vein (PV) remains unobstructed, thus mimics the high pressures achieved by systemic hydrodynamic injections in mice. this approach resulted in high efficient liver transduction with minimal toxicity and long-term gene expression [37]. More recently, an improved minimally invasive balloon occlusion method was reported to achieve high efficiency hepatocyte transduction using clinically relevant low HDAd doses (Figure 3). In this method, a single sausage shaped balloon was inflated in the inferior vena cava (IVC) to occlude the hepatic venous outflow and the HDAd was injected directly into the liver via the hepatic artery resulting in up to 80-fold higher levels of transgene expression compared to the systemic administration which persisted for more than 2.5 years [38]. Acute toxicity associated with this method of HDAd delivery was mild to moderate and transient.

Alternative strategies to attenuate the innate immune response are currently under investigation. One potential approach to reduce the visibility of the Ad vector to the immune system is the alteration of the capsid surface by virion encapsidation with cationic liposome [104] which resulted in 70–80% decrease in serum cytokines compared to unencapsidated virions without compromising the hepatic transduction efficiency. Another approach consists in the administration of PEGylated Ad vectors which resulted in a 50% to 70% decrease in serum cytokine compared to un-PEGylated Ad [105–107]. In a recent study the combination of methylprednisolone, an anti-inflammatory glucocorticoid, and PEGylated Ad potently inhibited IL-6 elevation [108]. Finally, it has been reported that a single administration of dexamethasone, another anti-inflammatory glucocorticoid, prior to Ad administration was able to significantly reduce both innate and adaptive immune response [109].

### 5.2. Gene therapy of Cystic Fibrosis lung disease

Cystic fibrosis (CF) is the most common life-limiting autosomal recessive disease, and it is estimated that 1 in 2500 Caucasian newborns are affected with CF although higher frequencies have been found in inbred population groups such as the Hutterites in Alberta, Canada (incidence of 1 in 313), Afrikaners in South Africa (1 in 355) and French Canadians (1 in 895) [110]. CF is a life-threatening disease and is characterized by chronic lung infections and inflammation that results in a life expectancy of 30–35 years [111]. The CF gene, termed CFTR (cystic fibrosis transmembrane conductance regulator), is a chloride channel expressed on the apical side of the airway epithelial cells. The lack of CFTR in CF patients causes a defect in water and ions exchange through the airway epithelium resulting in formation of thick mucus and subsequent lung inflammation and infection.

Gene therapy strategies are highly desirable and attractive for CF because it is a monogenic disease with the main pathology in the lung, which is relatively easy to access for treatment. Theoretically, one single administration of a gene delivery vector expressing CFTR could be effective in restoring the protein activity and cure the disease.

**Figure 4.**
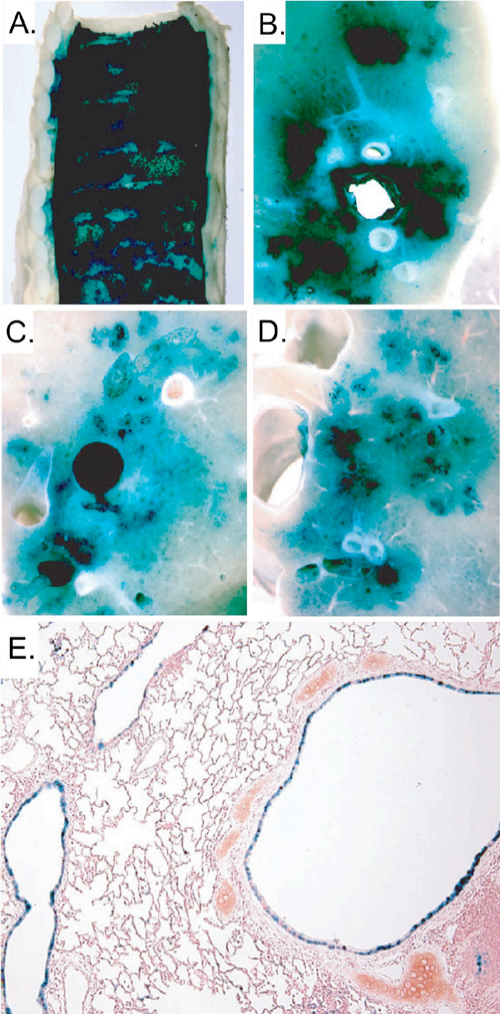
Pulmonary transduction in rabbits following intratracheal aerosolization of L-a lysophosphatidylcholine and HDAd expressing LacZ under the control of the K18 promoter. X-gal stained **(A)** trachea, **(B)** right upper lobe, **(C)** left lower lobe, **(D)** right lower lobe and **(E)** bronchus and bronchioles.

Several methods and strategies have been investigated and Ads are amongst the most utilized vectors. In fact, FGAds have been extensively pursued for the treatment of CF in animal models as well as in humans. Unfortunately, they have shown to be inadequate, having numerous disadvantages and serious shortcomings. The first obstacle encountered was the absence of the CAR receptors on the apical surface of the airway epithelial cells, which constitutes a barrier to adenovirus-mediated gene delivery *in vivo*. It was discovered that the CAR resides on the basolateral surface of the airway epithelial cells and that the tight junctions prevent the interaction between Ad and its receptor, precluding efficient transduction [112,113]. Another drawback that is associated with the use of FGAd is the adaptive immune response elicited by the residual viral gene expression from the vector backbone. Indeed, pulmonary delivery of FGAd in non human primates and human results in a dose-dependent inflammation and pneumonia [114–117]. In order to overcome the barrier represented by the absence of apical receptors, several strategies have been pursued based on the transient disruption of tight junctions. It has been shown that pre-treatment with EGTA, EDTA, polycations and other agents [118–120] is able to relax the tight junction and improve the delivery of Ad vectors into airway epithelia. To overcome the cytotoxicity and the adaptive immune response against FGAds, multiple deleted Ads have also been used. These vectors were able to reduce but not eliminate the inflammation and pneumonia which is likely due to a leaky expression of the viral late genes [121,122]. In contrast to FGAds, HDAds have shown to be very promising for the treatment of CF. Toietta *et al.* showed that HDAd delivered to the airway of mice resulted in an absence of pulmonary inflammation and the duration of transgene expression persisted at least for 15 weeks [5]. The studies with HDAd vectors have also shown that the human cytokeratin 18 promoter (K18) is expressed, similarly to the mouse CFTR, in the epithelium of the large airways and bronchioles and in sub-mucosal glands with a little expression in alveoli. Specifically, one study with HDAd encoding CFTR under K18 control delivered to CFTR-/- mice lung, showed the correct localization of the CFTR protein in the appropriate target cell types and the protection of the lungs from opportunistic infections [123]. This latter feature suggests that HDAd has the potential to reduce the susceptibility to opportunistic pathogens in CF patients. However, to successfully deliver the vector to the correct cell types in the above experiments, Koehler *et al*. pretreated CFTR-/- mice with EGTA to open the tight junctions. Despite the encouraging results obtained in these experiments, the requirement for two separate administrations, one for EGTA and one, 30 minutes later, for the HDAd vector, is suboptimal in terms of safety and efficacy. Indeed, to fully take advantage of EGTA pre-treatment, both the vector and EGTA need to be delivered in the same location, which is not guarantee in the case of two independent administrations. This issue was addressed by Koehler *et al.* [124] in another study in which they demonstrated the superior efficacy of HDAd vector formulated with 0.1% L-α-lysophosphatidylcoline (LPC). This specific formulation of the vector permitted one single efficient administration of HDAd along with the tight junction opening agent. However the intranasal delivery of the vector as performed in mice (spontaneous liquid inhalation) is not clinical relevant for larger animal and humans. To overcome this obstacle, Koehler *et al.* [124] used an intracorporeal nebulizing catheter (Aeroprobe) to aerosolize the HDAd-LPC solution directly into the trachea and lungs of rabbits. They showed that the HDAd administered in this way was able to achieve high level of transgene expression in the proximal and distal airway epithelium, from the trachea to terminal bronchioles (Figure 4). This strategy of delivering HDAd to rabbits has recently been applied to non human primates with similar encouraging results [125].

Disruption of tight junctions is clearly effective for increasing transduction of the airway and this treatment is well tolerated in animal models. Indeed, repeated aerosolization of EDTA into CF patients resulted in no harmful effects [126]. However, it would be highly desirable if this intervention could be avoided. Thus, development of modified HDAd able to transduce the airway epithelium via the apical surface would be very attractive. It should be pointed out that the aforementioned studies were performed in animal models with healthy airways and that transduction will likely be reduced in the lungs affected by multiple bacterial colonizations and thick mucus such as the human CF lungs. Up to now, efficacy of gene therapy has been only addressed in animal models with unaffected airways such as the CFTR knock-out mice and the nonhuman primates. The recently developed pig model for CF could potentially provide a better model for assessing the efficacy of experimental treatments in the CF lung disease [127]. However, several strategies can be envisioned to address this obstacle in the clinical setting. For example, severely affected CF patients may undergo commonly employed regimens to clear their lungs before gene transfer. This could include inhaled antibiotics (such as Tobramycin) and systemic intravenous anti-pseudomonal antibiotics (such as aminoglycosides, beta lactams, fluoroquionoes), pulmonary treatment with mucolytic agents (such as Pulmozyme), along with mechanical airway clearance to reduce the amount of mucus. Conducting gene transfer in CF patients with less affected lungs may be an alternative option, including the enrollment of younger CF patients with little or no lung disease. While somewhat controversial, this is not without precedence. Indeed, in a recent clinical trial using AAV, CF patients as young as 12 years of age were enrolled [128]. .In summary, while the thickened mucus remains a barrier for all gene transfer vectors (viral or nonviral) as well as for small molecule therapeutics we do not believe it to be insurmountable, especially considering the low levels of gene transfer that may be required which has been estimated to be 5 to 10% [129].

### 5.3. HDAd for muscle-directed gene therapy

Duchenne Muscular Dystrophy (DMD) is an X-linked lethal disorder that affects 1 in 3500 male births and is caused by genetic mutation in the dystrophin gene. The protein dystrophin is an essential structural component of the skeletal muscle cell membrane and its deficiency results in instability of the muscle cell and fiber degeneration. Since there is a lack of effective treatments, gene therapy strategies aimed at transferring normal copies of the dystrophin gene into the muscle fibers of patients, appears one of the most desirable options to cure this disease. The cDNA for full-length dystrophin is approximately 14 kb, far above the size of most of gene therapy vectors [130]. HDAd has opened the possibility to treat DMD animal models due to its large cloning capacity (up to 37 kb) which can accommodate up to two copies of the dystrophin gene [131,132]. HDAds expressing full-length dystrophin gene have been shown to restore the full dystrophin-glycoprotein complex in the skeletal muscle. In these studies, neonate skeletal muscles of mdx mice (a model for DMD) injected with HDAd were able to express dystrophin for the duration of the experiment (up to one year) resulting in the amelioration of the pathogenesis of the disease and in a reduced level of muscle degeneration with functional correction of muscle contractility [131]. However a significant inflammatory response was been observed which was accompanied by humoral response against the murine dystrophin protein expressed by transduced muscle. This immune response may also occur in humans, as many DMD patients have a large dystrophin gene deletion. Therefore the dystrophin encoded by gene transfer vectors may be seen as a foreign antigen with the consequent development of an adaptive immune response and loss of long-term phenotypic correction. One potential strategy to bypass the immunity against the protein is the co-delivery of immunomodulatory molecules able to blunt the innate and adaptive immune response. Jiang *et al*. demonstrated that blockade of the costimulatory interaction between naïve T cells and antigen-presenting cells by co-delivering CTLA4Ig alone or in combination with CD40Ig, diminishes innate and adaptive immunity induced by HDAd-dystrophin and prolong the transgene expression [4,133,134]. HDAd have also been explored for *in utero* gene therapy for DMD. The immaturity of the fetal immune system accompanied by the survival advantage of the muscle cells expressing dystrophin over the dystrophin-deficient fiber, makes this approach very attractive. The application of this strategy *in vivo*, showed that HDAd is less toxic compared to FGAd and capable of driving stable transgene expression and restoration of the sarcoglycan complex [135]. In order to be an effective treatment for this disease, dystrophin needs to be expressed by multiple muscles, including the diaphragm because respiratory dysfunction is a main cause of death among DMD patients [136–138]. Therefore, diaphragm-directed gene therapy has been investigated in mdx mice with HDAd which leads to reversal of functional abnormalities of dystrophic diaphragms for at least 30 days [138].

The muscle is a very attractive target for gene transfer because, like the liver, it can be used as a cell factory for production and secretion of therapeutic proteins. In fact, skeletal myofibers constitute about 40% of the total body mass, have a relatively long half-life and can be easily transduced *in vivo* because of easy access. High seroprevalence of pre-existing anti-Ad neutralizing antibody in the adult human population represents an obstacle for intravenous delivery, and it has been shown that this could be minimized by local delivery of the vector. Interestingly, when HDAd was injected intramuscularly (i.m) into previously immunized mice, stable transgene expression could be achieved; in contrast, the same mice injected i.m. with FGAd lost transgene expression after three week [139]. This study also showed that even though it is possible to administer HDAd into pre-immunized mice, a 30- to 100 fold higher dose (compared to naïve mice) was required to achieve 87% and 100% transduction of the muscles.

Finally, another hurdle that hampers muscle-directed gene therapy with Ad vectors is that mature muscle is not transduced efficiently because of low level of CAR receptor expression on the surface of adult muscle cells. Bramson L. *et al*, showed that the incorporation of polylysine into the H-I loop of the adenoviral fiber protein can improve HDAd transduction of mature muscle cells, giving up to 21- fold increase compared to the unmodified counterpart [140].

### 5.4. Gene therapy for brain and eye

Because of their intrinsic ability to infect post-mitotic cells and to mediate long-term transgene expression, Ad vectors constitute a very promising gene-delivery platform for central nervous system (CNS) disorders [141]. The above features are critical in order to successfully treat disorders ranging from simple monogenic disorders (such as Lesh-Nyhan syndrome, leukodistrophies, lysosomal storage diseases, amyotrophic lateral sclerosis among others) to multifactorial diseases including Parkinson’s disease and Alzeimer’s disease.

Following systemic administration of an FGAd vector, a rapid decline in transgene expression has been observed in peripheral organs whereas the same FGAd vector is able to stably transduce adult brain cells [142–144]. Indeed, intraparenchymal injection of FGAd vectors into the brain elicits a minimal, transient local inflammation which does not compromise the duration of transgene expression. One possible explanation for this phenomenon is the “immune-privileged” status of the brain, being relatively protected from the effect of the immune response. In fact, Ad injections into the brain result in an ineffective T cell response against brain-transduced cells in presence of viral protein expression from the backbone of FGAd vectors [145,146]. However, the immune system can respond to antigenic stimuli in the brain if the host organism has pre-existing immunity against that antigen, which would be the case for pre-immunization or re-administration with the same vector. In this case, loss of transgene expression and chronic inflammation are observed following FGAd injection into the brain [147]. In contrast, injection of HDAd into the brain of pre-immunized mice does not show these detrimental effects. Instead, HDAd was able to mediate significantly higher levels of transgene expression with substantially reduced immune response [147,148]. Recently, interesting results have been reported by delivering HDAd into the cerebrospinal fluid through a lumbar puncture in primates [149]. In this study, it was shown that injection of an HDAd vector by lumbar puncture into the cerebrospinal fluid (CSF) of non-human primates allows long-term (three months) transduction of neuroepithelial cells. This result was also observed in monkeys bearing a pre-existing anti-adenoviral immunity [149]. Another study from the same authors showed that by using the same route of administration in immune-competent mice, it was possible to deliver HDAd expressing anti-inflammatory cytokines and achieve long term transgene expression without any signs of toxicity [150]. This latter strategy makes HDAd-CSF gene delivery a very attractive therapeutic approach for brain inflammatory condition such as multiple sclerosis.

Encouraging results have been obtained in a study in which stereotactic injection of HDAd expressing a short hairpin RNA to silence the Huntington disease gene was able to inhibit Huntington protein aggregation [151,152]. However, the vector had limited brain distribution not extending beyond a few millimeters from the needle track, making this approach still far from optimal. The brain is a complex organ with intricate interconnections between various cell types and therefore it could be challenging to develop a targeting strategy with HDAd for the correction of diseases with diffuse involvement. Nevertheless, diseases requiring localized gene delivery to a discrete set of neurons such as Parkinson’s disease or brain tumors may be more suitable.

HDAd vectors have recently emerged as an important therapeutic strategy for brain tumor treatment. In a preclinical study for the treatment of glioblastoma multiforme, intratumoral injection with HDAd encoding the conditionally cytotoxic herpes simplex type 1 thymidine kinase (TK) and the immunostimulatory cytokine fms-like tyrosine kinase ligand 3 (Flt3L) was associated with increased survival and development of antiglioma immunological memory without signs of neuropathology or systemic toxicity [153]. Given the high risk that FGAd treatment of glioblastoma multiforme can be compromised by prior exposure to natural Ad infection, HDAd vectors could offer a safer and more effective treatment for patients with this type as well other types of brain cancer.

There have been a limited number of studies investigating HDAd vectors for ocular gene therapy. In one study, HDAd vector was able to transduce and rescue cells from the neurosensory retina in a mouse model of retinal degeneration [154]. Moreover, HDAd vectors showed a great potential in targeting the retinal pigment epithelium following subretinal injection, without evidence of adverse immune reactions [155].

### 5.5. Ex vivo gene therapy in human patients

HDAd has recently been used in a clinical trial to treat anemic chronic kidney disease (CDK) patients [159]. In this Phase I–II study, a small number of autologous dermal fibroblasts were removed from under the skin of anemic CDK patients under local anesthesia and transduced ex vivo with an HDAd expressing erythropoietin (EPO). Following transduction, the amount of EPO produced by the transduced cells was measured so that the precise number of transduced cells could be reimplanted subcutaneously to achieve the requisite dose of EPO. No adverse events were reported in this trial and, importantly, elevated hemoglobin levels were sustained for up to one year after a single treatment with the HDAd transduced cells. Significantly, this study also clearly demonstrates that HDAd can be manufactured under cGMP.

### 5.6. HDAd as genetic vaccines

FGAds have been developed to express antigens and have proven to be valuable genetic vaccines. Recent studies have shown that HDAd may be superior than FGAd for this application. For example, Harui *et al*. [156] compared the ability of FGAd and HDAd expressing β-galactosidase to generate an immune response in mice and found that HDAd generated a stronger T cell and antibody response against β-galactosidase than FGAd. Weaver *et al*. [157] also found that HDAd-based vaccines generated stronger immune responses against the encoded antigen than FGAd-vaccines in mice. In addition, administration of HDAd–based vaccines resulted in lower tissue damage and anti-Ad T cell responses than FGAd. Weaver *et al*. [157] also demonstrated that HDAd induced anti-HIV immune response in rhesus macaques. In a subsequent study, Weaver *et al*. [158], demonstrated that rhesus macaques vaccinated with HDAd expressing HIV-1 envelope protected the animals from subsequent mucosal SHIV challenge. Because most humans are seropositive for adenovirus serotype 5, HDAd vaccines based on serotype 5 may be minimally, if at all, effective. To overcome this, Weaver *et al*. used HDAd vaccines based on serotypes 1, 2 and 6 and showed that the presence of pre-existing immunity to adenovirus serotype 5 in both the mice and rhesus macaques did not prevent successful vaccination [157,158]. These studies demonstrate the potential utility of HDAd as a genetic vaccine.

## 6. Concluding remarks and future perspectives

HDAd possess many characteristics that make them attractive vectors for gene transfer of a wide variety of applications. However, a major concern regarding clinical application of HDAd is the host innate inflammatory response against the vector capsid that occurs shortly after administration. This concern is primarily problematic for intravascular delivery as it does not appear as severe for other routes of administration. This innate immune response is multifactorial and its mechanism(s) remains largely unknown although some of the components involved are being progressively identified. Given the complexity of this reaction, it will be challenging to design strategies to minimize or reduce the severity of this response. Moreover, the fundamental differences among species make it difficult to predict the outcome in humans based on the result of the preclinical animal testing. Regardless of the multiple mechanisms involved, strategies to improve the transduction efficiency using lower vector doses are clinically attractive because the innate response is dose-dependent. Another problem is that most humans have pre-existing immunity to serotype 5 adenovirus. Since most current HDAd are based on serotype 5, concerns regarding reduced efficacy and increased toxicity in the face of pre-existing immunity must be considered. Possible solutions to this problem include PEGlyation of the vector or using HDAds derived from different, less common serotypes or even HDAd derived from non-human adenoviruses. We are optimistic that these barriers will be surmountable as research into their solutions is ongoing.
